# The effects of concurrent increases in supplementation of calcium and phytase on growth performance, balance of Ca and P, and bone mineralization in nursery pigs

**DOI:** 10.1093/tas/txad122

**Published:** 2023-11-08

**Authors:** Hengxiao Zhai, Jon Bergstrom, Jingcheng Zhang, Wei Dong, Zhenzhen Wang, Kostas Stamatopoulos, Aaron J Cowieson

**Affiliations:** DSM (China) Animal Nutrition Research Center, Bazhou 065799, China; DSM Nutritional Products, Parsippany, NJ 07054, USA; DSM (China) Animal Nutrition Research Center, Bazhou 065799, China; DSM (China) Animal Nutrition Research Center, Bazhou 065799, China; DSM (China) Animal Nutrition Research Center, Bazhou 065799, China; DSM Nutritional Products, 4303 Kaiseraugst, Switzerland; DSM Nutritional Products, 4303 Kaiseraugst, Switzerland

**Keywords:** bone, calcium, digestibility, phosphorus, phytase

## Abstract

The objective of this study was to investigate the effects of concomitantly increasing supplementation of Ca and phytase on growth performance, balance of Ca and P, and bone mineralization in nursery pigs. There were eight experimental diets. The positive control (PC) one and two were formulated to contain 0.64% and 0.85% total Ca, respectively, whereas the dietary concentrations of other nutrients were identical and adequate. The negative control (NC) was deficient in total Ca (0.48%) and total P (0.41%). Five combinations of incremental levels of Ca and phytase (0.48% and 1,750 phytase units [FYT]/kg, 0.52% and 2,000 FYT/kg, 0.55% and 2,250 FYT/kg, 0.59% and 2,600 FYT/kg, and 0.63% and 3,000 FYT/kg) were added to the NC to establish the remaining five experimental diets. Each diet was fed to six pens of six pigs (three barrows and three gilts per pen). All diets contained 3 g/kg TiO_2_, and fecal samples were collected from each pen during the trial. In the end, one pig per pen was euthanized to collect the right tibia and urine in bladder. The results showed that the pigs of NC gained less weight, consumed less feed, and utilized feed less efficiently than their counterparts fed the PC and the treatments with phytase (*P* < 0.01). With increasing supplementation of Ca and phytase, there was a tendency for gain:feed to decrease (*P* < 0.10). There was a significant reduction in bone dry weight; and in percentages, as well as weights of bone ash, Ca, and P; in pigs of NC compared with pigs of PC1, PC2, or phytase treatments. In comparison to PC2, PC1 and phytase treatments resulted in a higher percentage of bone P and greater weights of bone ash, Ca, and P (*P* < 0.05). There was no significant effect of concurrent supplementation of Ca and phytase on bone mineralization. The NC had significantly lower apparent total tract digestibility (ATTD) of Ca and P, lower concentrations of digestible Ca and P, but a higher ATTD Ca/ATTD P ratio than PC1, PC2, or the phytase treatments. The averages of ATTD of Ca and P in treatments with phytase were significantly higher than PC1 or PC2 (*P* < 0.01). With increasing addition of Ca and phytase, the ATTD of P, digestible Ca and P, and the ATTD Ca/ATTD P ratio increased linearly (*P* < 0.05), which contrasted with a linear reduction in ATTD of Ca (*P* < 0.05). Meanwhile, there was a linear (*P* < 0.01) increase in the concentration of urinary Ca. In conclusion, increasing the dietary supplementation of phytase in conjunction with the increasing dietary Ca level increased the dietary ATTD Ca/ATTD P ratio without damaging the absorption of P in the current study. The higher ATTD Ca/ATTD P ratio did not improve the bone mineralization markedly and thus the extra Ca was voided through urine.

## Teaser

The adverse effect of increasing dietary Ca/P ratio on P absorption in pigs could be counterbalanced by a concurrent increase in the phytase dose.

## Introduction

Adverse effects of increasing dietary total Ca/total P ratios on growth performance and bone mineralization have been widely reported (Qian et al., 1996; Lagos et al., 2019; Becker et al., 2020). These adverse effects were attributed to the formation of insoluble Ca-P complexes in the gastrointestinal tract (Stein et al., 2011), a reduction in phytase efficacy (Qian et al., 1996), and/or the formation of insoluble phytate complex that is inaccessible for phytase hydrolysis (Selle et al., 2009). Reducing the dietary total Ca/total P ratio from 1.9 to 1.3 in a practical diet, however, did not improve the efficiency of phytase in releasing P (Létourneau-Montminy et al., 2010). In addition, the adverse effects are more likely to be exacerbated when dietary P supply is deficient (Reinhart and Mahan et al., 1986) and to be ameliorated when dietary P supply is adequate (González-Vega et al., 2016; Lagos et al., 2019).

Increasing addition of Ca/limestone in a diet appears to cause a general reduction in P digestibility, but its effect on bone mineralization depends on the dietary P level. Qian et al. (1996) reported a linear decrease in apparent total tract digestibility (ATTD) of P with increasing total Ca/total P ratio from 1.2 to 2.0 in weaner pigs fed diets supplemented with phytase, which paralleled the adverse effects on growth performance and bone characteristics. In our previous study, increasing total Ca/total P ratio from 0.6 to 1.3 decreased ATTD of P, but increased bone mineralization in weaner pigs supplemented with phytase because the pigs conserved enough P through reduction of P excretion through urine to compensate for the decreased digestible P from diet (Zhai et al., 2023). In these studies, a fixed dose of phytase was supplemented. Increasing phytase supplementation along with an increase in dietary Ca/limestone level could maintain a constant supply of standardized total tract digestible (STTD) P from diet despite an increase in the ratio of STTD Ca to STTD P as indicated by our models predicting the release of STTD Ca and P from phytase based on the dietary concentrations of phytate, phytase, and Ca. This practice could be a potential alternative to increasing dietary P inclusion by at least 10% to 15% to avoid the strong negative impact of the higher STTD Ca to STTD P ratios on growth performance when maximizing bone ash compared with maximizing growth of pigs as recommended by Lee et al. (2023).

The aim of this study, therefore, was to investigate the effects of increasing dietary total Ca/total P ratio and phytase dose on the growth performance, digestibility of Ca and P, bone mineralization, and concentrations of Ca and P in urine and plasma in nursery pigs. We hypothesized that increasing phytase dose would counterbalance the negative effect of increasing dietary Ca level on P absorption and, thus, the bone mineralization will be improved with the increasing digestible Ca/digestible P ratios that result from the concurrent supplementation of phytase and Ca.

## Materials and Methods

This study was conducted at DSM (China) Animal Nutrition Research Center Co. Ltd. (Bazhou, P. R. China) with its protocol approved by the Animal Welfare Committee of DSM (China) Animal Nutrition Research Center (AWCCAN).

### Animals and Facilities

Two hundred and eighty-eight barrows and gilts (PIC L1050 × L337; initial body weight [BW**]** 7.9 ± 0.6 kg [mean ± standard deviation]) were used in a randomized complete block design. The pigs were weaned at ~21 d of age and transferred to a nursery facility for an adaptation period of 7 d. In the nursery facility, each pen (space/pen = 3.0 × 1.8 m^2^) had a plastic-coated wire floor and was equipped with two water nipples and one stainless-steel feeder. Prior to the trial, the pigs were individually weighed and allotted into 48 pens based on their initial BW and gender (three barrows and three gilts per pen). The pens in each BW block were randomly assigned to the dietary treatments, resulting in six replicate pens per dietary treatment. The experimental diets were fed for 21 d. Feed and water were supplied ad libitum. At the end of trial, the pigs weighed 19.1 ± 1.9 kg.

Room temperature and ventilation were controlled by a computer system to provide an optimal environment. The room temperature was 27 °C at the start and gradually reduced to 23 °C at the end. The relative humidity ranged from 45% to 75%.

### Experimental Diets

The ingredient and nutrient composition of the experimental diets are presented in Table 1. There were eight experimental diets. The positive control (PC) one and two were formulated to 0.37% STTD P, and 1.08 and 1.42 total Ca to total P ratios, respectively, whereas the dietary concentrations of other nutrients were identical and adequate. The negative control (NC) was deficient in Ca (0.48% total Ca) and P (0.20% STTD P) by reference to the recommendations for 7–11 and 11–25 kg pigs (NRC, 2012). Five combinations of incremental levels of Ca and phytase (0.48% and 1,750 phytase units [FYT**]**/kg, 0.52% and 2,000 FYT/kg, 0.55% and 2,250 FYT/kg, 0.59% and 2,600 FYT/kg, and 0.63% and 3,000 FYT/kg) were added to the NC to establish the remaining five experimental diets. These five diets were predicted by our models to provide the same concentration of STTD P as the PC diets considering the contribution from phytase in the presence of 0.25% phytate P, but their STTD Ca to STTD P ratios increased from 1.20 to 1.60 in comparison to 1.25 and 1.60 in PC1 and PC2. The lower ratio was supposed to maximize growth performance, whereas the higher one was to maximize bone ash. Our models include one for predicting STTD P release and the other one for STTD Ca release from phytase based on dietary levels of phytate, Ca, and phytase. Limestone was included at the expense of rice hulls to create different dietary Ca levels. Phytase (HiPhorius, DSM Nutritional Products, Switzerland) was added based on its analyzed phytase activity. Titanium dioxide was included at 3 g/kg feed as an indigestible marker to enable the measurement of ATTD of Ca and P. All diets were pelleted with a conditioning temperature of 75 °C.

**Table 1.** Ingredient and nutrient composition of the experimental diets (%, as-is basis)

Items	PC 1	PC 2	NC	Dietary Ca/phytase, %/FYT^1^				
				0.48/1,750	0.52/2,000	0.55/2,250	0.59/2,600	0.63/3,000
Ingredients								
Corn	58.34	58.34	58.34	58.34	58.34	58.34	58.34	58.34
Soybean meal	34.05	34.05	34.05	34.05	34.05	34.05	34.05	34.05
Soybean oil	2.50	2.50	2.50	2.50	2.50	2.50	2.50	2.50
NaCl	0.45	0.45	0.45	0.45	0.45	0.45	0.45	0.45
NaHCO_3_	0.15	0.15	0.15	0.15	0.15	0.15	0.15	0.15
DL-Met	0.12	0.12	0.12	0.12	0.12	0.12	0.12	0.12
L-Lys·HCl	0.45	0.45	0.45	0.45	0.45	0.45	0.45	0.45
L-Thr	0.15	0.15	0.15	0.15	0.15	0.15	0.15	0.15
L-Val	0.10	0.10	0.10	0.10	0.10	0.10	0.10	0.10
Limestone	0.90	1.47	0.75	0.75	0.85	0.95	1.05	1.16
Monocalcium phosphate	1.12	1.12	0.25	0.25	0.25	0.25	0.25	0.25
Vitamin–mineral premix^2^	0.50	0.50	0.50	0.50	0.50	0.50	0.50	0.50
Rice hulls^3^	0.57	0.00	1.59	1.59	1.49	1.39	1.29	1.18
TiO_2_	0.30	0.30	0.30	0.30	0.30	0.30	0.30	0.30
Benzoic acid	0.30	0.30	0.30	0.30	0.30	0.30	0.30	0.30
Total	100.00	100.00	100.00	100.00	100.00	100.00	100.00	100.00
Calculated nutrients and energy								
Metabolizable energy, kcal/kg	3,347	3,347	3,347	3,347	3,347	3,347	3,347	3,347
Crude protein	21.8	21.8	21.8	21.8	21.8	21.8	21.8	21.8
Standardized digestible Lys	1.29	1.29	1.29	1.29	1.29	1.29	1.29	1.29
Phytate P	0.25	0.25	0.25	0.25	0.25	0.25	0.25	0.25
Total Ca^4^	0.64	0.85	0.48	0.48	0.52	0.55	0.59	0.63
Total P^4^	0.60	0.60	0.41	0.41	0.41	0.41	0.41	0.41
Total Ca/total P	1.08	1.42	1.16	1.16	1.25	1.33	1.42	1.52
Standardized digestible P^4^	0.37	0.37	0.20	0.37	0.37	0.37	0.37	0.37

^1^Phytase was premixed with 5 kg of corn to ensure its uniform distribution in each diet; NC, negative control; PC, positive control.

^2^Premix supplied per kilogram of diet: vitamin A, 9,750 IU; vitamin D_3_, 3,000 IU; vitamin E, 63 mg; vitamin K_3_, 3.0 mg; vitamin B_1_, 3.0 mg; vitamin B_2_, 9.6 mg; vitamin B_6_, 4.5 mg; vitamin B_12_, 36 μg; D-biotin, 240 ug; D-calcium pantothenate, 30 mg; folic acid, 1.8 mg; niacin, 36 mg; Cu (tribasic copper chloride), 115 mg; I (potassium iodate), 0.6 mg; Fe (ferrous sulfate), 120 mg; Mn (manganese sulfate), 60 mg; Zn (zinc sulfate), 120 mg; Se (sodium selenite), 450 μg; choline (choline chloride), 300 mg; and Ca (calcium carbonate) 0.6 g.

^3^Nutrients and energy were calculated without considering the contribution of rice hulls.

^4^Total Ca and total P did not consider phytase contribution, but the standardized digestible P included P release from phytase.

### Measurement and Sampling

The pigs were individually weighed on days 0 and 21 of trial, and the feed consumption per pen was recorded during the trial to calculate average daily gain, average daily feed intake, and gain:feed.

Fresh and clean fecal samples were grabbed from each pen on days six to eight of trial. Existing feces in each pen were removed before collection on each collection day. A total of ~500 g of fresh feces was collected per day per pen. All the fecal samples collected from each pen during the 3-d collection period were pooled and mixed to homogeneity with a hand-held blade mixer (TD-110, RuiBao Hardware Co. Ltd., Dongguan, P. R. China). A sub-sample of ~500 g for each pen was stored at −20 °C until further processing.

Blood, the right tibia, and urine from the bladder were collected from the pig in each pen with BW closest to the pen average on day 21 of trial. Urine was aspirated from the bladder (Foury et al., 2005), but the measurements were not corrected for creatinine. Blood samples were collected from the vena cava into heparin vacutainer collection tubes and immediately centrifuged at 3,000 × g for 10 min to obtain plasma. All the samples were stored at −20 °C before processing. The tibias were processed according to the non-defatting bone processing procedures described by Wensley et al. (2020). In short, the bones were autoclaved at 120 °C for 30 min to facilitate the removal of muscular tissues and cartilaginous caps. The cleaned bones were left at room temperature for 1 d and then oven-dried at 105 °C for 7 d. Finally, the dried tibias were incinerated in a muffle oven for 72 h at 600 °C.

### Chemical Analyses

The fecal samples were oven-dried to a constant weight and ground to pass through a 0.5-mm screen before analysis. The dietary and fecal samples were dried at 105 °C in an oven for 4 h for dry matter determination (method 934.01; AOAC International, 2006). Titanium, Ca, and P were determined by Inductively Coupled Plasma-Optical Emission Spectrometry (ICP-OES; Optima TM 8000, PerkinElmer, Shelton, USA; method 985.01; AOAC International, 2006) after microwave digestion. Urine samples (10 mL) were dried at 60 °C before the microwave digestion. Plasma Ca and P were analyzed on a chemistry analyzer (AU480, Beckman Coulter, Brea, USA). The phytase activity was determined by colorimetric measurement of the released phosphate from phytate. One phytase unit was defined as the amount of enzyme that releases 1 µmol of inorganic phosphate from 50 mM phytate per minute at 37 °C and pH 5.5. These analyses were performed in duplicate, except that Ca, P, and phytase activity in the feed samples were determined from three replicates.

### Calculations and Statistical Analyses

The experiment was a randomized complete block design. Each pen or pig was an experimental unit. ATTD of Ca and P, and ATTD Ca and P were calculated as described by Zhai et al. (2023).

The data were analyzed using the MIXED procedure of SAS 9.4 (SAS Inst. Inc., Cary, NC) with the model including the dietary treatment as a fixed effect, replicate as a random effect, and an error term. Polynomial orthogonal contrasts were constructed to test the linear and quadratic effects of increasing dietary Ca and phytase levels and to compare among treatments of NC, PC, and phytase. The least-square means were presented, and the significance was defined at *P* < 0.05. A trend was declared at *P* < 0.10.

## Results

### Experimental Diets and the Analyses

There was good agreement between the formulated and analyzed Ca and P concentrations and phytase activities in experimental diets. Analyzed dietary Ca levels and phytase activities were within 5% of their target values (Table 2). Analyzed dietary P levels were ~7% higher than the formulated values, and thus the analyzed dietary Ca/P ratios (1.04 to 1.48) were slightly lower than the expected values (1.08 to 1.52).

**Table 2.** Analyzed calcium and phosphorus content (as-is basis, %) and phytase activity (FYT/kg) of diets

Diet^1^	Ca	P	Ca/P ratio	Phytase
PC 1 (Ca/P, 0.64%/0.60%)	0.65	0.63	1.04	48
PC 2 (Ca/P, 0.85%/0.60%)	0.84	0.64	1.31	67
NC (Ca/P, 0.48%/0.41%)	0.49	0.45	1.09	52
Ca/phytase, %/FYT				
0.48/1,750	0.48	0.44	1.10	1,822
0.52/2,000	0.51	0.44	1.17	2,038
0.55/2,250	0.56	0.44	1.28	2,301
0.59/2,600	0.59	0.44	1.35	2,668
0.63/3,000	0.65	0.44	1.48	3,003

^1^NC, negative control; PC, positive control.

### Growth Performance and Bone Mineralization

The pigs fed the NC gained less weight, consumed less feed, and utilized feed less efficiently than their counterparts fed the PC diets and the dietary treatments with phytase (*P* < 0.01; Table 3). The phytase treatments, on average, and PC1 showed an increase in pig’s final BW, average daily gain, and feed efficiency compared to PC2 (*P* < 0.01). The average daily feed intake of the pigs fed the phytase treatments tended to be higher than that of the pigs with PC2 (*P* = 0.05). With increasing supplementation of Ca and phytase, there was a tendency for gain:feed to decrease (*P* < 0.1).

**Table 3.** Growth performance of the pigs fed diets with increasing supplementation of Ca and phytase^1^

Diet^2^	IBW^2^, kg	FBW^2^, kg	ADG^2^, g/d	ADFI^2^, g/d	Gain:feed, g/kg
PC 1 (Ca/P, 0.64%/0.60%)	7.9	19.5	553	731	757
PC 2 (Ca/P, 0.85%/0.60%)	7.9	18.7	511	704	726
NC (Ca/P, 0.48%/0.41%)	7.9	16.8	422	646	654
Ca/phytase, %/FYT					
0.48/1,750	7.9	19.4	550	728	756
0.52/2,000	7.9	19.5	551	722	764
0.55/2,250	7.9	19.4	546	736	742
0.59/2,600	7.9	19.6	559	746	749
0.63/3,000	7.9	19.5	550	744	741
SEM^3^	0.01	0.24	12	14	7
*P* value					
NC vs. PC1	0.85	<0.01	<0.01	<0.01	<0.01
NC vs. PC2	0.91	<0.01	<0.01	<0.01	<0.01
NC vs. Ca/phytase	0.70	<0.01	<0.01	<0.01	<0.01
PC 1 vs. PC2	0.76	0.02	0.02	0.18	<0.01
Ca/phytase vs. PC1	0.89	0.88	0.88	0.79	0.40
Ca/phytase vs. PC2	0.59	<0.01	<0.01	0.05	<0.01
Ca/phytase					
Linear^4^	0.97	0.78	0.80	0.21	0.05
Quadratic^4^	0.58	0.99	0.99	0.97	0.94

^1^There were six replicates per diet treatment.

^2^NC, negative control; PC, positive control; IBW, initial body weight; FBW, final body weight; ADG, average daily gain; ADFI, average daily feed intake.

^3^SEM, standard error of the mean.

^4^Linear and quadratic effects of increasing supplementation of Ca and phytase.

There was a significant reduction in bone dry weight, and in the percentages and weights of bone ash, Ca, and P, for pigs fed NC compared with pigs fed PC1, PC2, or the phytase treatments (Table 4). The bone Ca/P ratio was significantly higher in pigs of NC than that of PC1 and the average of phytase treatments. The average of phytase treatments and PC1 resulted in a higher percentage of bone P and greater weights of bone ash, Ca, and P in comparison to PC2 (*P* < 0.05). Additionally, when compared with PC2, the percentages of bone ash and Ca were significantly higher in pigs of PC1, and the dry bone weight and percent bone Ca tended to increase in pigs fed the phytase treatments.

**Table 4.** Bone mineralization of the pigs fed diets with increasing supplementation of Ca and phytase^1^

Diet^2^	Bone dry weight, g	Bone ash, %	Bone Ca, %	Bone P, %	Bone ash, g	Bone Ca, g	Bone P, g	Bone Ca/P ratio
PC 1 (Ca/P, 0.64%/0.60%)	12.5	49.0	17.3	8.9	6.1	2.2	1.1	1.95
PC 2 (Ca/P, 0.85%/0.60%)	12.3	45.8	16.3	8.3	5.6	2.0	1.0	1.96
NC (Ca/P, 0.48%/0.41%)	8.8	36.3	12.7	6.4	3.2	1.1	0.6	1.98
Ca/phytase, %/FYT								
0.48/1,750	12.5	47.3	16.8	8.6	5.9	2.1	1.1	1.95
0.52/2,000	13.2	46.8	16.4	8.5	6.2	2.2	1.1	1.92
0.55/2,250	13.3	47.7	16.9	8.7	6.3	2.2	1.1	1.95
0.59/2,600	12.8	47.7	17.2	8.7	6.1	2.2	1.1	1.97
0.63/3,000	13.0	47.7	16.9	8.7	6.2	2.2	1.1	1.94
SEM^3^	0.3	0.9	0.3	0.1	0.2	0.06	0.03	0.01
*P* value								
NC vs. PC1	<0.01	<0.01	<0.01	<0.01	<0.01	<0.01	<0.01	0.04
NC vs. PC2	<0.01	<0.01	<0.01	<0.01	<0.01	<0.01	<0.01	0.14
NC vs. Ca/phytase	<0.01	<0.01	<0.01	<0.01	<0.01	<0.01	<0.01	<0.01
PC 1 vs. PC2	0.66	0.01	0.02	<0.01	0.03	0.04	0.03	0.52
Ca/phytase vs. PC1	0.22	0.11	0.20	0.16	0.86	0.71	0.65	1.00
Ca/phytase vs. PC2	0.08	0.10	0.07	0.03	<0.01	<0.01	<0.01	0.40
Ca/phytase								
Linear^4^	0.55	0.52	0.29	0.37	0.26	0.20	0.21	0.41
Quadratic^4^	0.18	1.00	0.99	0.83	0.17	0.19	0.21	0.72

^1^There were six replicates per diet treatment.

^2^NC, negative control; PC, positive control.

^3^SEM, standard error of mean.

^4^Linear and quadratic effects of increasing supplementation of Ca and phytase.

### Digestibility of Ca and P, and Digestible Ca and P, in Experimental Diets,

The NC had lower ATTD of Ca and P, lower concentrations of digestible Ca and P, but a higher ATTD Ca/ATTD P ratio than PC1, PC2, or the phytase treatments (*P* < 0.01; Table 5). The digestible Ca and the ATTD Ca/ATTD P ratio were significantly higher in PC2 than in PC1 despite a significant reduction in the ATTD of Ca. The averages for ATTD of Ca and P in the treatments with phytase were significantly higher compared with PC1 or PC2, and the averages of the digestible Ca and the ATTD Ca/ATTD P ratios were higher with the phytase treatments than PC1, but less than PC2 (*P* < 0.01).

**Table 5.** Apparent total tract digestibility (ATTD) of Ca and P in experimental diets with increasing supplementation of Ca and phytase^1^

Diet^2^	ATTD of Ca, %	ATTD of P, %	Digestible Ca, g/kg	Digestible P, g/kg	Digestible Ca/P ratio
PC 1 (Ca/P, 0.64%/0.60%)	64.5	53.9	4.21	3.39	1.24
PC 2 (Ca/P, 0.85%/0.60%)	61.7	53.7	5.18	3.44	1.51
NC (Ca/P, 0.48%/0.41%)	52.8	36.5	2.58	1.64	1.58
Ca/phytase, %/FYT					
0.48/1,750	80.3	76.1	3.87	3.35	1.16
0.52/2,000	79.8	78.2	4.07	3.41	1.19
0.55/2,250	79.7	77.7	4.48	3.40	1.32
0.59/2,600	79.1	78.0	4.66	3.41	1.37
0.63/3,000	77.9	78.1	5.06	3.43	1.48
SEM^3^					
*P* value					
NC vs. PC1	<0.01	<0.01	<0.01	<0.01	<0.01
NC vs. PC2	<0.01	<0.01	<0.01	<0.01	<0.01
NC vs. Ca/phytase	<0.01	<0.01	<0.01	<0.01	<0.01
PC 1 vs. PC2	0.02	0.71	<0.01	0.15	<0.01
Ca/phytase vs. PC1	<0.01	<0.01	<0.01	0.79	<0.01
Ca/phytase vs. PC2	<0.01	<0.01	<0.01	0.12	<0.01
Ca/phytase					
Linear^4^	0.03	0.01	<0.01	0.02	<0.01
Quadratic^4^	0.48	0.10	0.34	0.39	0.36

^1^There were six replicates per diet treatment.

^2^ATTD, apparent total tract digestibility; NC, negative control; PC, positive control.

^3^SEM, standard error of mean.

^4^Linear and quadratic effects of increasing supplementation of Ca and phytase.

With increasing addition of Ca and phytase, the ATTD of P, digestible Ca and P, and the ATTD Ca/ATTD P ratio increased linearly (*P* < 0.05), which contrasted with a linear reduction in ATTD of Ca (*P* < 0.05).

### Concentrations of Ca and P in Plasma and Urine

In plasma, the concentration of P was significantly lower in NC than in PC1, PC2, or the phytase treatments (Table 6). The concentration of P in PC1 tended to be higher than in PC2 and the phytase treatments (*P* < 0.1). The plasma concentration of Ca of PC2 was significantly higher than that of PC1 and tended to be higher than the average of the phytase treatments (*P* = 0.06).

**Table 6.** Concentrations of calcium and phosphorus in plasma and urine of pigs fed diets with increasing supplementation of Ca and phytase^1^

Diet^2^	Plasma Ca, mg/dL	Plasma P, mg/dL	Urine Ca, mg/L	Urine P, mg/L
PC 1 (Ca/P, 0.64%/0.60%)	11.5	9.0	294	28
PC 2 (Ca/P, 0.85%/0.60%)	12.1	8.1	1481	13
NC (Ca/P, 0.48%/0.41%)	11.7	3.8	1417	9
Ca/phytase, %/FYT				
0.48/1,750	11.4	8.8	102	18
0.52/2,000	11.8	8.5	206	26
0.55/2,250	11.6	8.4	632	17
0.59/2,600	11.8	8.0	1010	11
0.63/3,000	11.6	8.3	793	10
SEM^3^	0.2	0.3	202	7
*P* value				
NC vs. PC1	0.46	<0.01	<0.01	0.08
NC vs. PC2	0.19	<0.01	0.86	0.65
NC vs. Ca/phytase	0.81	<0.01	<0.01	0.33
PC 1 vs. PC2	0.04	0.06	<0.01	0.13
Ca/phytase vs. PC1	0.48	0.08	0.30	0.15
Ca/phytase vs. PC2	0.06	0.45	<0.01	0.58
Ca/phytase				
Linear	0.50	0.13	<0.01	0.33
Quadratic	0.26	0.44	0.43	0.74

^1^There were six and five replicates per diet treatment for plasma and urine minerals, respectively.

^2^NC, negative control; PC, positive control.

^3^SEM, standard error of mean.

^4^Linear and quadratic effects of increasing supplementation of Ca and phytase.

In urine, the concentration of P tended to be lower in NC than in PC1 (*P* = 0.08). The concentration of Ca in PC2 and NC were significantly higher than that in PC1 and the average of phytase treatments. With increasing supplementation of Ca and phytase, there was a linear (*P* < 0.01) increase in the urinary concentration of Ca.

## Discussion

A deficiency of P can result in a poor appetite and decreased growth in pigs (Jongbloed, 1987; NRC, 1998). In this study, P deficiency as exemplified by NC caused a reduction in both feed intake and feed utilization efficiency. The depressed feed intake indicates a loss of appetite attributable to the inadequate P supply. As verified in broilers, dietary P level regulates appetite through modulation of gut and hypothalamic expression of anorexigenic genes (Aderibigbe et al., 2022). The poor feed efficiency impaired protein deposition because of the need for P as an initiation factor for protein synthesis that may be affected by P deficiency. Moreover, it is well-recognized that there is a close relationship between whole-body P and whole-body N content in pigs (NRC, 2012) given that protein deposition and bone development are disconnected in finishing pigs (Lautrou et al., 2020). As expected, a recovery in both feed intake and feed utilization efficiency occurred when either monocalcium phosphate or phytase was added to the P-deficient diet.

Phosphorus absorption can be reduced by wide dietary Ca/P ratios, with consequences of poorer growth and bone calcification (NRC, 1998). An excess supply of Ca from the diet can lead to formation of an insoluble Ca-P complex in the gut and decreased availability of P (Stein et al., 2011). The detrimental effect on P availability was also attributed to a reduction in phytase efficacy (Qian et al., 1996; Liu et al., 1998). However, Driver et al. (2005) pointed out that the margin of improvement by phytase supplementation was greater for animals fed diets with wide Ca to P ratios, so the adverse effect of wide dietary Ca/P ratios might have been due to insufficient P to meet requirements. In this study, the ATTD P in PC1 and PC2 were measured to be very close to the recommendations by NRC (2012), but could be marginally deficient because the actual P requirements were greater than NRC (2012) recommendations (Vier et al., 2019a; b). The negative impact of additional Ca on growth performance is more common when feeding P-deficient diets (González-Vega et al., 2016). However, an increase in Ca supply in PC2 relative to PC1 did not lead to a reduction in P digestibility or in the concentration of ATTD P, but BW gain and feed efficiency were still impaired. Meanwhile, a tendency for poorer feed efficiency was observed in pigs fed the diets with the combined addition of limestone and phytase even though the measured dietary ATTD Ca/ATTD P ratio increased in tandem with a slight increase in the concentration of ATTD P. Therefore, there might be other reasons, besides the interaction between Ca and P, that are involved with reduced growth performance in association with extra Ca in the diet. An interaction between Ca and other nutrients could be implicated. For example, an interaction between Ca and fatty acids could result in the formation of insoluble calcium fatty acid soaps, which could reduce fat absorption and increase the fecal excretion of fat and energy as demonstrated in humans (Jacobsen et al., 2005) and rats (Govers and Van der Meer, 1993). This agrees with the improvements in the digestibility of some nutrients in pigs by a reduction in dietary Ca and P (Johnston et al., 2004), but the apparent ileal digestibility of protein and amino acids may not be affected by dietary Ca levels in pigs (Lee and Stein, 2022).

Adequate nutrition of Ca and P depends on an adequate supply of both elements at a suitable ratio (Peo, 1991). Poor bone mineralization was observed in pigs of both NC and PC2 relative to those of PC1. The NC diet was confirmed to be deficient in P by having less digested P when compared to the known requirement (NRC, 2012). A deficient P supply reduced absorption and retention of P, as well as Ca, and ultimately led to poor bone mineralization (Sørensen, et al., 2018, 2019). Both PC1 and PC2 had a similar concentration of ATTD P in line with NRC (2012) recommendations, but PC2 contained ~20% more digestible Ca. Therefore, the observed negative impact of extra Ca on bone mineralization in pigs of PC2 was not realized through a compromise in P absorption. This contradicts the notion that an excessive supply of Ca reduces P digestibility and impairs growth performance and skeletal development (NRC, 1998; Stein et al., 2011; Becker et al., 2020). In response to the extra Ca load, the pigs of PC2 gave a lower digestibility of Ca and a higher urinary concentration of Ca, which indicates that the pigs adapted to absorb less Ca from gut and to excrete more Ca through urine. The increased urinary excretion of Ca (1.19 g/L urine) by pigs of PC2 compared to that of PC1 pigs could have led to a lower retention of Ca in consideration of their smaller difference in the ATTD Ca (5.18 vs. 4.21 g/kg feed). This implies a strong need/capacity of pigs to maintain Ca homeostasis, which aligns with the tighter regulation of blood Ca level than blood P level (Vötterl et al., 2021), and the smaller variability in plasma Ca level than in the plasma P level in the current study. In addition, the pigs of PC2 showed a higher plasma concentration of Ca, but a lower plasma concentration of P, which agrees with the well-recognized inverse relationship between serum concentrations of Ca and P as dietary Ca and P increased (Maxson and Mahan, 1983). It is noteworthy that the ionized Ca in blood may be more physiologically meaningful than total Ca under certain environments (Forman and Lorenzo, 1991).

The coordinated supplementation of phytase and limestone resulted in increased dietary ATTD Ca/ATTD P ratios as expected, but bone mineralization was not improved. The dietary P level in the current study could have limited bone mineralization as indicated by the minimal urinary P excretion. Urinary P excretion increases above the basal level only when dietary P supply exceeds the physiological needs (Grez-Capdeville and Crenshaw, 2021). The extra Ca appeared to be excreted through urine, which indicates that the assayed tibias had been saturated with Ca. Increasing dietary Ca will increase the amount of retained P until bone tissues reach a plateau (Létourneau-Montminy et al., 2012). The lack of differences in bone responses to concurrently increasing dietary Ca and phytase contradicts the damaging effects on bone mineralization that were associated with adding extra Ca alone to pigs of PC2 when compared with pigs of PC1. These differences may be related to varied intensity of the hormonal responses triggered by different Ca supplies. The pigs of PC2 showed a higher concentration of Ca in both plasma and urine than the pigs fed phytase diets. The greater elevation in the plasma concentration of Ca could have elicited a stronger hormonal regulation that resulted in excessive urinary excretion of Ca and a depression in bone resorption. Additionally, the supplemental phytase can alter the timing of Ca and P appearance in blood and their coordination in bone deposition (Cowieson et al., 2011, 2017). The lack of significant improvement in bone mineralization with increasing addition of limestone and phytase prompted us to consider that the ATTD Ca/ATTD P ratio of 1.16 was already adequate for the pigs at the ATTD P concentration of 3.4 g/kg feed. However, some numerical improvements in most bone parameters continued up to the ATTD Ca/ATTD P ratio of 1.32. This ratio was equivalent to a STTD Ca/STTD P ratio of 1.30 after correcting for the basal endogenous losses of Ca and P, which is close to the optimal STTD Ca/STTD P ratio of 1.35 in 11 to 25 kg pigs recommended by González-Vega et al. (2016). The basal endogenous losses of Ca and P were assumed to be 190 mg/kg dry matter intake based on the estimates by Baker (2011) and González-Vega et al. (2013). The recommended STTD Ca/STTD P ratio of 1.67:1 to maximize bone ash in 11 to 22 kg pigs when the diet contained 0.33% STTD P (Lee et al., 2023) appeared to be too high considering the reduced bone mineralization in pigs of PC2 at an ATTD Ca/ATTD P ratio of 1.51 (equivalent to a STTD Ca/STTD P ratio of 1.48) and an ATTD P level of 0.34%.

In our previous study (Zhai et al., 2023), increasing dietary Ca/P ratio reduced ATTD of P and the concentration of digestible P in diets supplemented with 1,000 FYT/kg feed. In consideration of phytase as something with a potential counterbalance to the negative effects of high dietary Ca on P absorption, we hypothesized that the P digestibility and the digestible P content in a diet will not be impaired if we increase the dose of phytase as we increase the supplementation of Ca. This hypothesis was found to be true in this experiment because both the P digestibility and the concentration of the dietary digestible P increased with concurrently increasing supplementation of limestone and phytase. Moreover, the measured ATTD P concentrations in phytase diets (3.35 to 3.43 g/kg feed) were very similar to those in PC diets (3.39 to 3.44 g/kg feed), attesting to the precision of our prediction models. The greater increase in dietary ATTD Ca (3.87 to 5.06 g/kg feed) relative to that in ATTD P (3.35 to 3.43 g/kg feed) with increasing supplementation of Ca and phytase resulted in an increase in the ATTD Ca/ATTD P ratio as we expected. Following those expectations, we also hypothesized that an increase in digestible Ca/digestible P ratio would improve bone calcification. However, this hypothesis was proven to be false because no significant change was observed in bone calcification.

## Conclusion

In conclusion, increasing the dietary supplementation of phytase in coordination with the supplementation of Ca increased the dietary ATTD Ca/ATTD P ratio without damaging P absorption in the current study. The higher ATTD Ca/ATTD P ratios did not improve bone mineralization markedly, which could have been limited by the dietary P level as indicated by the minimal urinary P excretion, and thus the extra Ca was voided through urine.
